# National Egg Scandal

**Published:** 1916-04-22

**Authors:** 


### "NATIONAL EGG SCANDAL."

Under this heading *Truth* of the 12th instant deals with the National Egg Collection for the Wounded, of which the patron is H.M. Queen Alexandra. It sets forth that this scheme has met with "the approval and grateful appreciation of the War Office," while from all parts of the country poultry owners have been packing and dispatching eggs for use in hospitals in this country and in France literally in millions. The scheme was hatched by the *Poultry World*, 154 Fleet Street, which is owned by the Poultry Press, Limited, of which a Mr. F. Carl is the managing editor. After going into the circumstances of the formation of the organisation and the causes which have led Mr. Bates, the proprietor of *Poultry*, who failed to obtain a full balance-sheet with details of salaries, travelling expenses, advertising, stamps, and other items, which he properly held were owed as a duty to the public, who had by gifts of eggs and money made so notable a success of the fund, and the proprietors of the *Feathered World* to retire from the scheme, *Truth* proceeds as follows:—

I am given to understand that at the end of the first financial year—*i.e.*, in November 1915—the National Egg Collection took credit in round figures for 12,000,000 eggs supplied to hospitals. In cash the public contributions amounted to nearly £5,000. In addition to this sum the committee had been supplied with £33,000 of public money by subvention from the War Office. Adding the two cash items together it will be seen that every egg supplied to a wounded soldier cost ¾d., an extravagantly high charge for the mere collection of the eggs, especially in view of the fact that freight charges are not taken into account. The organisers of the collection may, therefore, very well fear being called to account for extravagance. But the fear of criticism is justification neither for a public department nor a private committee refusing to furnish the public with details of their dealings with public funds, whether obtained from the taxpayers or benevolent contributors. If the War Office goes into the charity business it must conform to the conditions expected of every other charity promoter and administrator or take the consequences. Meanwhile, there is fortunately a means by which the suppressed accounts may be made public. It will be seen from the letter printed in *Truth* that the accounts prepared by the chairman of the committee are in possession of the War Office. It is, therefore, open to any member of Parliament to move for their publication, and by so doing place the public in a position to judge from the way it has already dealt with £38,000 whether the committee of the National Egg Collection can be wisely entrusted with the further expenditure of public money.

We have received a request for support this year for this egg collection from the office of the *Poultry World*, 154 Fleet Street, which we cannot support pending the satisfactory settlement of the matters set forth. How many of those familiar with the offices of the *Poultry World* before this collection was started have inspected them within recent days?

